# Structural aspects of plasticity in the nervous system of *Drosophila*

**DOI:** 10.1186/s13064-018-0111-z

**Published:** 2018-07-01

**Authors:** Atsushi Sugie, Giovanni Marchetti, Gaia Tavosanis

**Affiliations:** 1 Center for Transdisciplinary Research, Niigata University, Niigata, 951-8585 Japan; 2 Brain Research Institute, Niigata University, Niigata, 951-8585 Japan; 3 Center for Neurodegenerative Diseases (DZNE), 53127 Bonn, Germany

**Keywords:** Structural plasticity, *Drosophila*, Photoreceptors, Synapse, Active zone, Mushroom body, Mushroom body calyx, Learning

## Abstract

Neurons extend and retract dynamically their neurites during development to form complex morphologies and to reach out to their appropriate synaptic partners. Their capacity to undergo structural rearrangements is in part maintained during adult life when it supports the animal’s ability to adapt to a changing environment or to form lasting memories. Nonetheless, the signals triggering structural plasticity and the mechanisms that support it are not yet fully understood at the molecular level. Here, we focus on the nervous system of the fruit fly to ask to which extent activity modulates neuronal morphology and connectivity during development. Further, we summarize the evidence indicating that the adult nervous system of flies retains some capacity for structural plasticity at the synaptic or circuit level. For simplicity, we selected examples mostly derived from studies on the visual system and on the mushroom body, two regions of the fly brain with extensively studied neuroanatomy.

## Background

The establishment of a functional neuronal circuit is a dynamic process, including an extensive structural remodeling and refinement of neuronal connections. Intrinsic differentiation programs and stereotypic molecular pathways contribute the groundwork of patterning the nervous system during development, including the guidance of axons and dendrites over long distances or the recognition of appropriate connection partners. In addition, information derived from functional cues controls the refinement of the circuit. Even after the major task of assembling a fully functional network has been achieved, the nervous system retains the capacity of undergoing not only functional, but also structural modifications related, for instance, to adaptation or learning. The role of activity in the developmental refinement of neuronal morphology and of the connections within a circuit (and possibly also the setting up of circuits; [1]) as well as in the initiation of structural remodeling during adult life is undisputed [2]. In intricate neuropils, dense with axons and dendrites of different neuronal types, the feed-back derived from activity appears to be an important element to define which connections can be stabilized and which ones removed [3–5]. Nonetheless, the cellular mechanisms initiated by activity to drive structural remodeling during development and in the course of adult life are not fully elucidated. Here, we review the literature supporting structural plasticity in the fruit fly *Drosophila*, a system offering major advantages for genetic and molecular analysis. Where appropriate, we include comparisons with other invertebrate and vertebrate systems to highlight evolutionary conserved mechanisms. Thanks to the stereotypy of the “macroscopic” organization of the fly’s nervous system, work carried out using *Drosophila* led to major breakthroughs in the identification of conserved molecular cascades and mechanisms that orchestrate genetically controlled developmental programs. Possibly due to this emphasis on stereotypy, the role of signals providing feed-back information about functional connections during fly nervous system development has not been investigated as deeply. Nonetheless, multiple examples of activity controlling neuronal complexity during development have emerged [6]. For instance, dendrite elaboration of fly larval motorneurons as well as of the wide-field serotonergic neuron CSDn in the *Drosophila* central nervous system can be affected by the level of input signals and actually by input activity during development [7, 8]. Similarly, exposure of the larva to different light regimes modifies the total dendrite length of ventral lateral neurons (LNv), postsynaptic to the photoreceptors [9]. The accessibility of the neuromuscular junction (NMJ) of larvae allows for detailed molecular, morphological and functional analysis [10]. The level of activity in the motorneuron can modulate the number of boutons formed and the density of synaptic release sites at the NMJ, providing a clear example of activity-related structural control [11–13]. In this context, postsynaptically-derived signals carried by the Wnt and BMP signaling pathways, modulate the presynaptic terminal at the NMJ [14–16].

Evidence for structural rearrangements in the nervous system of the adult fly after development is completed has been rather limited and it is mostly related to adaptive phenomena. As an example, prolonged exposure to a given odor induces increased size and synaptic density in discrete glomeruli of the antennal lobe, the first olfactory processing center [17, 18]. Nonetheless, the behavior of adult flies (as well as of larvae) can be modified by experience in a non-adaptive fashion. In fact, flies can learn multiple types of cues and form lasting memories, a capacity that might require structural modifications in the neurons and the circuits involved [19–21].

Recent large-scale efforts are yielding complete maps at synaptic-resolution of circuits within the adult fly central nervous system, including areas involved in memory formation [22, 23]. This information can be combined with the availability of tools to visualize, manipulate and control the activity of restricted and defined populations of neurons in this system [24–27]. Thus, novel insights to the fundamental understanding of information processing and of learning are starting to be produced and much more is expected in the coming years [22, 28–30]. Importantly, the high-resolution description of circuits obtained in electron microscopy images and with tools to highlight synaptic components is challenging the idea of circuit stereotypy in the fly nervous system. As an example, the detailed study of motorneuron network in the ventral nerve cord of the larva revealed a high degree of variability in terms of synaptic connections [31].

Taken together, it appears that it is the right time to approach the non-stereotypy and plasticity of neurons in the adult fly nervous system.

For the purpose of this review, we define structural plasticity as the changes that include physical remodeling of recognizable structures. In particular, we concentrate on large-scale changes that might involve neuronal processes, their connections and circuit subroutines and on molecular changes that affect particularly the structural organization of the presynapse. Certain types of functional plasticity involve structural changes, as the formation of new dendritic spines [32] or the reorganization of the molecular components of the synapse [33, 34]. In this review we will select the aspects that deal in particular with the structural components of functional and synaptic plasticity. We chose to focus on two centers of the fly nervous system to summarize the current evidence in support of an influence of activity during development and of plastic changes in the adult nervous system in adaptive or learning conditions.

## The establishment of circuits

The ease of manipulating their input makes sensory systems particularly suitable for the study of activity-dependent processes involved in neuronal circuit assembly, refinement and plasticity. In this review we concentrate therefore our attention on the fly adult visual system and on the pathways that deliver olfactory information to the mushroom body (MB), involved in memory processing.

**Fig. 1:**
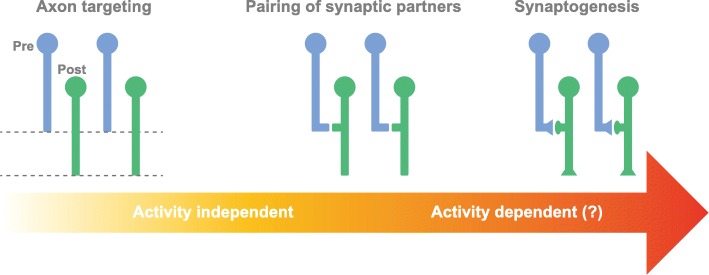
Activity dependent modulation of neuronal connectivity during development in the *Drosophila* visual and MB circuits. Steps supporting the establishment of neuronal circuits in the adult *Drosophila* visual and olfactory systems during development

Sensory information is initially encoded in discrete stereotypic pathways. For instance, the presence of a bright signal in the visual field or the specific odorant present in the air flux activates defined subroutines within the visual or olfactory circuits, respectively. To maintain the initial specificity of information and to transmit it precisely towards higher processing centers, circuits are assembled with remarkable precision during development. Correct axon and dendrite targeting to the appropriate region, pairing of the suitable synaptic partners and synaptogenesis are all highly regulated developmental steps (Fig. 1). In principle, targeting and recognition of processes to form functional connections can be achieved through genetically defined pathways. For instance, specific tags and receptors allow the correct partner neurons to recognize each other. Alternatively, guidance signals could support the formation of initially sloppy maps, which are subsequently refined. In this case, the evaluation of the functional performance of a given connection or of the circuit is likely to be a highly valuable factor for deciding whether the connection should be maintained or removed [35].

Here, we address how much these two potential mechanisms contribute to the assembly of circuits in the visual system or in the MB. While the extant literature regarding the molecular mechanisms of genetically controlled programs is abundant, particularly for the visual system, the information about activity-dependent circuit assembly control is rather scant in *Drosophila*. We put our emphasis primarily on this second, less explored aspect.

### The initial connectivity in the visual system is independent of activity

**Fig. 2:**
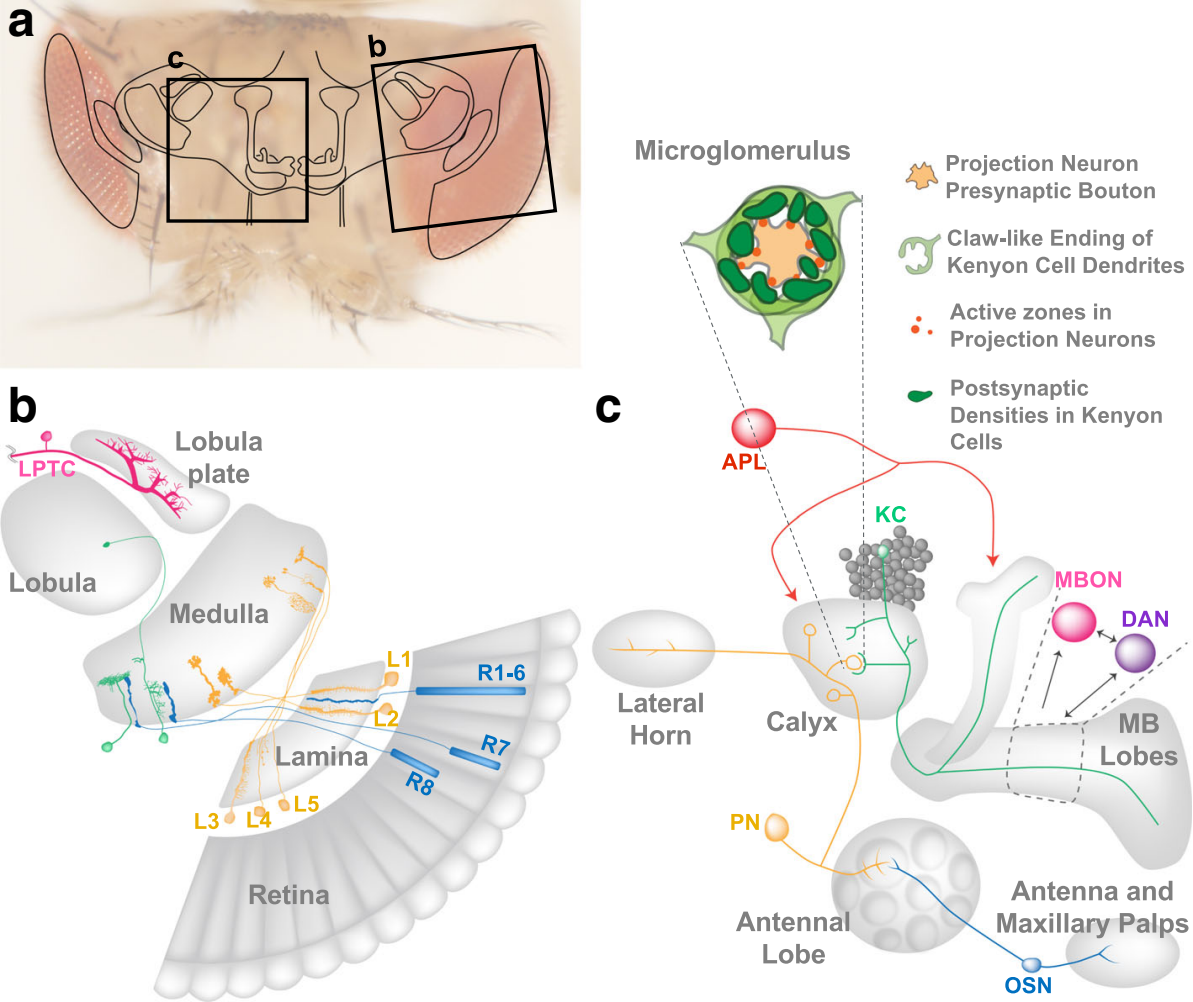
Visual system and olfactory circuit in the adult fly brain. **a** Dorsal view of the adult *Drosophila* head and schematic drawing of major brain centers, including the visual system and the MB (boxes). **b** Horizontal section of the visual system including the retina, lamina, medulla, lobula and lobula plate. Representative neuron types cited in this review are shown, including photoreceptors (blue; R1–6, R7 and R8), lamina neurons (orange; L1-L5), medulla neurons (green) and a Lobula plate tangential cell (magenta; LPTC). **c** Schematic representation of the pathways delivering olfactory information to the MB*.* Olfactory sensory neurons (OSN) in the antennae and maxillary palps send axons to specific glomeruli in the antennal lobe (AL), where they form synaptic contacts with projection neurons (PNs). PNs convey olfactory sensory input to the lateral horn and to the calyx of the mushroom bodies (MB). In the MB calyx PN axonal projections and MB dendrites create synaptic complexes, named microglomeruli (MG). MB neurons process the olfactory information by integration of signals of anterior paired lateral neuron (APL) and dopaminergic neurons (DANs) to control mushroom body output neurons (MBONs)

Light is received in the *Drosophila* compound eye by photoreceptors and is transmitted to three optic ganglia in the visual circuit (Fig. 2a and b) [36]. There are ~ 780 ommatidia in the retina, each containing eight photoreceptors (R1 to R8). R1–6 project into the first optic ganglion, the lamina, while R7 and R8 project their axons to the M6 and M3 layers, respectively, of the medulla, the second optic ganglion. The five subtypes of lamina neurons (L1 to L5) project into distinct layers in the distal medulla (Fig. 2b). Within the third optic ganglion, the lobula complex, the lobula plate tangential cells (LPTCs) integrate information from R1–6 to compute the direction of optic flow (Fig. 2b) [37].

The pairing of appropriate synaptic partners is an essential aspect for the establishment of functional circuits (Fig. 1). How much of this recognition is driven by genetic programs as opposed to functional cues in the visual system? As a striking example of circuit assembly controlled by recognition among identity tags, each different synaptic pair in the medulla expresses distinct Immunoglobulin superfamily cell adhesion molecules (21 Dprs and 9 DIPs) for precise synaptic partner matching [21, 38, 39]. Once appropriate partners have come to close proximity, cell adhesion molecules contribute to synaptic formation among them [40]. Interestingly, also functional components of the presynaptic active zone (AZ) such as DLiprin-α and DSyd-1 are required not only for synaptic vesicle (SV) clustering at R7 axon terminal synapses, but also for axon targeting [41, 42]. These data are intriguing as they suggest a negative relationship between synapse assembly and axon extension. Taken together, cell surface molecule diversity contributes to axon targeting, pairing of synaptic partners and synaptogenesis, suggesting a robust genetically controlled program supporting these events.

Activity-dependent fine-tuning of neuronal circuits plays a role during the development of the visual system in vertebrates [43–45]. Several studies addressed whether neuronal activity is relevant for precisely assembling neuronal circuits in the *Drosophila* visual system as well. They provide evidence that neuronal circuit formation is independent of neuronal activity in the visual system, especially for the photoreceptors R1–6 [46]. The number of synapses in R1-R6 and the downstream circuit organization has been investigated in a series of neuronal activity mutants, including Phospholipase C *norpA* [47] and Ca^2+^ channels *trp* and *trpl* [48, 49] mutants that suppress the generation of electropotentials, or histidine decarboxylase *hdc* [50, 51] and the Ca^2+^ sensor synaptotagmin *syt*^*AX4*^ [52] mutants that inhibit neurotransmitter release. All those mutants show no obvious defect in R1-R6 axon targeting or in the number of presynaptic AZs in the lamina [46]. Also deeper in the visual system, the complexity of LPTC dendrites is not affected by a constant darkness (DD) regime. In addition, LPTC dendritic spine structure and density remain unchanged after genetically-induced visual deprivation elicited by the expression of *head involution defective* (*hid*) in the eye [53]. From these studies, axon projection or dendrite arborization in the fly visual system seems to be defined largely independently of activity (Fig. 1).

### Contribution of experience to larval visual system connectivity

Although activity seems dispensable for the establishment of connectivity in the *adult* visual system, recent work points to its involvement of activity within *larval* visual circuits to guarantee the establishment of correct morphologies. The larval optic nerve, called Bolwig’s nerve (BN), projects into the central brain along a simple invariant path. The BN is required for the appropriate arborization of a serotonergic neuron and for the development of the dendritic tree of the circadian pacemakers, ventral lateral neurons (LN(v)s) [54, 55]. Suppression of synaptic activity in the presynaptic BN disrupts the dendritic arborization of the postsynaptic neurons in the larval visual system [56]. In this study, tetanus toxin light chain (TeTxLC), which blocks synaptic release by cleaving neuronal-Synaptobrevin, was expressed in photoreceptors leading to a reduction of the dendritic arborization of the serotonergic neuron. In contrast, attenuation of evoked activity by the expression of a genetically modified Shaker K^+^ channel (EKO channel) in photoreceptors did not alter the dendrites of this serotonergic neuron. While the possibility of a broader effect of Synaptobrevin inhibition remains, these results suggest that spontaneous synaptic activity could promote dendrite arborization in the serotonergic neuron. Also the arborization of the dendrites of the ventral lateral neurons LN(v)s at the third instar larval stage depends on activity. In fact, prolonged light exposure reduced, while constant darkness increased the LN(v) dendritic length via the cyclic adenosine monophosphate (cAMP) pathway [9]. Larvae are continuously exposed to sensory stimuli. Thus, experience might contribute to the adjustment of neuronal connectivity to guarantee appropriate synaptic strength in a variety of environments (Fig. 1).

### Activity-dependent development and maturation of the olfactory and mushroom body circuits

In insects, olfactory information is captured by olfactory sensory neurons (OSNs) on antenna and maxillary palps. OSNs project their axons to the antennal lobe (AL) that anatomically resembles the rodent olfactory bulb and represents the first center for olfactory information processing. The second-order olfactory projection neurons (PNs) convey the olfactory sensory input to the higher olfactory centers MB and lateral horn (LH) (Fig. 2c) [57]. The MBs are prominent paired neuropils implicated in higher order processing such as olfactory sensory integration, learning and memory, and spatial integration [58–60]. The core elements of the MBs are the Kenyon cells (KCs). Typically, KCs extend a single neurite that forms dendrite branches in the MB calyx and a single axon with terminal arborizations in the lobes (Fig. 2c) [61]. Olfactory information is delivered to the MB calyx by PNs that in the adult form specialized synaptic complexes, called microglomeruli (MG), with the dendrites of KCs (Fig. 2c) [62, 63]. Those connections are reminiscent of the mossy fibers to granule cells synapses in the cerebellum [64]. Interestingly, the PN to KC connectivity is not stereotyped and individual flies show distinct wiring patterns in the calyx [65, 66]. Other neuronal types, including modulatory ones, innervate the calyx. Among those, a large inhibitory anterior paired lateral (APL) neuron sends projections across the calyx, peduncle, and lobes (Fig. 2c) [67–69]. The output of approximately 2000 KCs per adult brain hemisphere converges onto a population of only 34 MB output neurons (MBONs) of 21 anatomically distinct types [27] (Fig. 2c).

Neural activity appears to be largely dispensable during metamorphosis for the establishment of the adult fly olfactory circuit [70]. For instance, the glomerular map in the *Drosophila* AL was not modified when all odor-evoked activity was eliminated or when input or output neurons were removed [71–73]. However, complementary work in social insects suggests that the presence and function of olfactory sensory neurons (OSNs) is fundamental for the development of the olfactory circuit. In particular, ants carrying mutations in the highly conserved co-receptor of odorant receptors (ORs) Orco, showed a striking reduction in the AL glomeruli number associated with deficiencies in social behavior [74]. Similarly, surgical removal of the antenna of honeybees at different time points during pupal development led to decreased synapse density in the AL in a stage-dependent manner [75]. Clearly, further studies are needed towards a comprehensive view of the role of neural activity in adult olfactory circuit wiring in insects.

After metamorphosis the adult fly emerges from the pupal case with a formed olfactory circuit. It appears nonetheless that the first days of adult life represent a critical period in which the olfactory circuit can undergo activity-dependent refinement. For instance, prolonged exposure to CO_2_ causes activity-dependent volume increase of the CO_2_-responding AL glomerulus. Those changes are reversible and occur in a critical time window corresponding to early adult life. In fact, exposure-induced plasticity in the CO_2_-responding glomerulus was not observed in flies 11 days post eclosion [18]. Whole-cell recordings of cultured MB neurons derived from late stage *Drosophila* pupae reveal spontaneous Ca^2+^ transients that might play a role in the maturation of the adult circuit [76]. At the molecular level, the RNA-binding protein Fragile X Mental Retardation Protein (FMRP) regulates MB circuit refinement in an activity-dependent manner [77]. FMRP is required at late pupal stages and during early adult life to control MB axonal pruning and presynaptic refinement in the MB calyx [77, 78]. Repressing PN activity during the first day after pupal eclosion results in enhancement of presynaptic axonal branching [78]. In addition, blocking PN synaptic vesicle release post-eclosion for 5 days yields increased bouton size [79]. Thus, the time following eclosion could represent a period in which the *Drosophila* olfactory circuit is evaluated and adapted to the local environment. Similar critical periods have been documented for the development of the mammalian cortex and olfactory bulb [80, 81]. In all these model systems, the critical period likely allows the animal to compare the developmentally determined network template with external conditions and make activity-dependent adjustments that reflect the external environment.

## Plasticity during adult life and ageing

Even after functional circuits have been established during development and refined during a critical period, they can still undergo structural and functional changes to allow the animal to adapt to a modified sensory environment or store relevant information to modify future behavior. While studies investigating functional plasticity in *Drosophila* have a long tradition, evidence for structural plasticity in the adult nervous system has been rather fragmentary.

### Structural plasticity in the adult visual system

Visual experience during early adult life can modulate behavior in *Drosophila*. In visually guided choice behavior tests, flies reared in darkness (DD) are attracted to wider vertical black lines against a white background compared to control flies reared in a regular light-dark cycle (LD), providing evidence for developmental visual plasticity in this system [82, 83]. DD reared flies also show lower preference for visible light in comparison to flies reared in an LD cycle in a Y-maze apparatus designed to test phototaxis preference behavior [84]. The plasticity of phototaxis preference is reversible in adult flies and can be modulated by the expression levels of N-methyl-D-aspartate receptor 1 (NMDAR1) [84]. Taken together, light exposure conditions during early adult life can modulate adult visual behavior, suggesting some plasticity in circuit function.

Defined patterns of activation of the presynaptic neuron can modulate synaptic function [85, 86]. The increase or reduction of activity at individual synapses is achieved via modifications of the postsynaptic response, for instance by modulation of neurotransmitter receptor availability at the postsynapse [87, 88]. Presynapses can also undergo plastic changes that ultimately modulate neurotransmitter release, as revealed by studies using rodent primary neuronal cultures [33, 89–91]. A recently described mechanism of presynaptic plasticity involves the modulation of the molecular composition of the synaptic vesicle (SV) release site at the presynaptic or active zone (AZ). At the AZ a conserved set of molecules of the ELKS/CAST, Rab3-interacting molecule (RIM), RIM-binding protein (RIM-BP), Liprin-α, SYD-1 and UNC-13 families coalesce to bring close together SVs and the voltage-gated Ca^2+^ channels that initiate their release [92, 93]. Prolonged silencing of cultured rat cortical neurons significantly decreases the expression levels of presynaptic proteins ELKS/CAST, RIM family Bassoon and Piccolo, Munc13, Liprin-α, and Synapsin to different extent [94]. In addition, activity blockade leads to recruitment of AZ machinery such as the voltage-gated Ca^2+^ channels in hippocampal neurons, revealed with super-resolution imaging [33]. In turn, the probability of neurotransmitter release correlates with the amount of Bassoon or RIM in rat or mouse neuronal cultures [94–96]. In this system, also the localization of Liprin-α2 at AZs depends on activity and the expression level of Liprin-α2 regulates the probability of SV release [97]. Taken together, these studies performed with rodent neurons indicate that the level of activity in the presynaptic neuron controls the abundance of AZ proteins, which in turn affects the probability of SV release at the synapse.

**Fig. 3:**
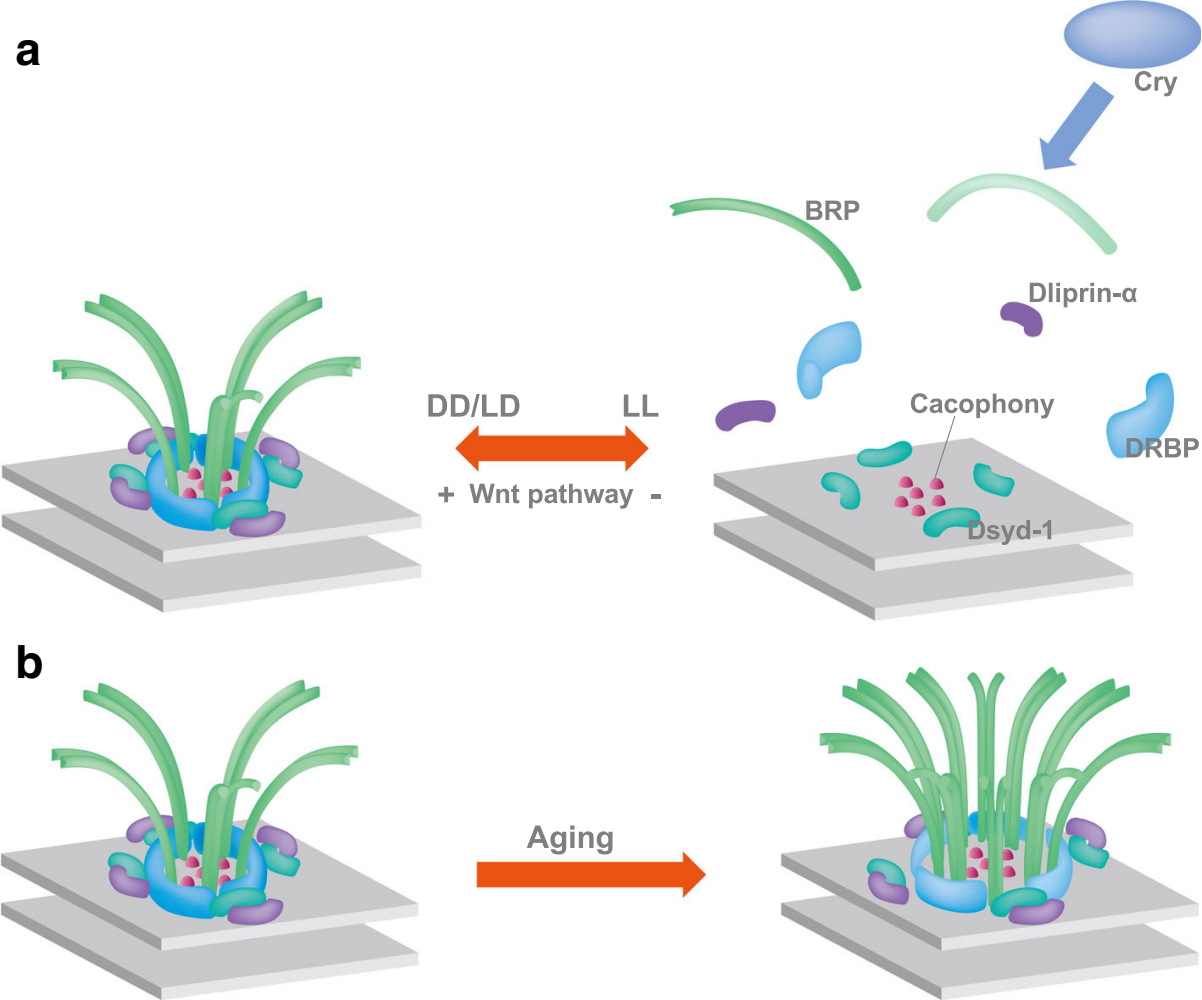
Environment-dependent modulation of synaptic components in the *Drosophila* visual and MB circuits. **a** Modulation of active zone components upon prolonged exposure to light. In constant darkness (DD) or in a light/dark cycle (LD), the divergent canonical Wnt pathway stabilizes the active zone structure. Constant light (LL) suppresses the divergent canonical Wnt pathway, leading to delocalization of BRP, DLiprin-α, and DRBP from active zone. Cryptochrome (Cry) forms a complex with BRP under light exposure. **b** Age-related structural changes in synapses of MB calyx. Ageing induces a consistent enlargement of the AZ associated with an increased number of BRP molecules

Notably, an activity-dependent remodeling of the AZ proteins has been described recently in the adult fly visual system. The presynaptic AZ in insects is marked by the presence of a T-shaped structure formed by the ELKS family Brp protein [98, 99] (Fig. 3). In spite of its complexity [10, 100, 101], the T-bar can dynamically disassemble and re-assemble. Rapid shifts from a dark regime to light or vice versa can induce reversible changes in the size and number of presynaptic T-bars in the photoreceptor neurons of the house fly (Musca domestica) within minutes [102]. The structural changes are reflected by measurable changes in protein abundance. Already a short light stimulation significantly increases the level of BRP, Synapsin and Dlg proteins in the *Drosophila* lamina even just for 15 min exposure to light [103]. In addition to these rapid changes, late-onset modifications alter synaptic composition by prolonged light exposure. A subset of AZ components as BRP, DLiprin-α, and DRBP are lost from the AZ in this condition, while DSysd-1 or the voltage-gated Ca^2+^ channel Cacophony is not (Fig. 3a). This presynaptic remodeling is triggered by a postsynaptic signal that elicits microtubule destabilization in the presynaptic photoreceptors via the divergent canonical Wnt pathway (Fig. 3a) [104, 105]. Only a subset of AZ undergoes these reversible modifications and loses their T-bar. Since loss of BRP suppresses transmission from the AZ [98, 99], it is expected that the final outcome of these changes is a reduction in transmission, potentially supporting homeostasis in the circuit. Taken together, light exposure can induce activity-regulated structural changes in the fly photoreceptor AZs.

### Structural plasticity in the mushroom body calyx

The MB has been most extensively studied in the context of associative memory that utilizes olfactory or other sensory information to guide future behavior [106–110]. Coincidence detection of sensory information (odor) and value (reward or punishment) initiating memory formation involves the MB output synapses and circuits at the lobes [111, 112]. The recurrent circuits between MBONS, dopaminergic neurons (DANs) and KCs contribute to memory consolidation [29] and to its re-consolidation after re-evaluation [30]. The contribution of additional circuits to multiple aspects of memory representations will require future investigations.

The MB calyx is involved in the extraction of a sparse code from the sensory information derived from the olfactory PNs [113, 114]. Electrophysiological recordings in PNs and KC upon odor exposure reveal that the MB transforms the flood of odor-elicited activation of the PNs into a sparse representation of the odor in the KCs [113, 115, 116]. This sparse format of sensory coding is a widely observed feature in vertebrate cortical areas suggesting that minimizing the overlap between representation patterns of different stimuli maximizes memory capacity [117]. Sparse KC activation is important for odor discrimination after associative olfactory learning [118]. Recently generated genetically-encoded functional reporters targeted to either pre- or postsynaptic compartments allow to monitor Ca^2+^ dynamics during olfactory processing in the adult fly brain. Imaging of odor-evoked activity of synapses in the MB calyx revealed experience-dependent changes in pre- and postsynaptic activity [79]. The reduced anatomical complexity and the ability to monitor physiological changes in identified neurons suggests that studies in the fly will likely deliver important contributions to the understanding of how activity-dependent functional plasticity is generated, reinforced and maintained in a neuronal circuit.

An additional major feature of the insect MB calyx is that it displays no obvious stereotypy. While subsets of PNs, project to loosely defined calycal regions [119, 120] the identity of their postsynaptic KCs cannot be foretold [66, 113]. KC subtypes project their dendrites also to approximate layers within the calyx [119–121]. Nonetheless, a single PN bouton is predicted to contact different types of KCs [63]. Although, it remains conceivable that the genetic tools available do not allow yet recognizing existing stereotypy, anatomical and functional data support the view that PN/ KC connections are not predefined [66, 113]. Which are the rules that control setting up such a non-stereotypical circuit remains an open and fascinating question.

Experience dependent structural plasticity in the MB calyx has been explored extensively in social insects such as bees and ants. Those studies indicate that the volume of the MB calyx is modulated by experience. The initial exploration of the foraging area by these insects correlates with an increase in MB calyx volume [122–125]. In honeybees, the experience-dependent volume increase of the MB calyx correlates with activity mediated by muscarinic cholinergic pathways [126]. The core functional unit of the MB calyx is the synapse formed by individual PN boutons and multiple KCs dendrite endings of claw shape (Fig. 2c) [62, 63, 127]. The resulting large synaptic complex, the microglomerulus (MG), also comprises input of additional extrinsic neurons that in *Drosophila* have not yet been unequivocally identified [63, 127]. MGs are readily detectable using antibodies recognizing presynaptic markers such as Synapsin in the PN bouton or by highlighting actin in the KC dendrites [128, 129]. Using such tools, it was possible to show that PN bouton size and postsynaptic densities increase during the behavioral transition of honeybees from nursing to foraging [130, 131]. Such findings suggest that the MGs might be a major component of MB calyx plasticity. Importantly, in bees the establishment of long-term olfactory associative memories correlates with an increase in the density of MGs, specifically in the calycal region responding to olfactory stimuli [132]. Collectively these data, together with comparable results obtained in other insect species [122, 133, 134], point to the MG as sites of structural plasticity related to experience and learning. Whether MGs size and number might be directly affected by experience or in learning has not been directly tested in *Drosophila*. However, MG properties can be modulated by input activity also in this system. The use of genetic tools to specifically label subsets of PNs and their presynaptic structures, in combination with postsynaptic markers expressed in KCs, allows to image MGs in the adult fly calyx at high resolution [135]. Prolonged deprivation of PN synaptic input in the adult MB calyx leads to increased MG number and enlarged pre and postsynaptic elements in the silenced MGs [79, 135]. These effects could represent a homeostatic response to decreased neuronal activity. They suggest that olfactory experience encoded by PN neuron activity induces MG structural changes [79, 135]. However, how functional plasticity in response to odor stimulation correlates with structural modifications remains to be tested.

Synapses within the adult fly calyx undergo age-related structural modifications, as recently shown with sophisticated tools originally developed for studies at the NMJ. While learning scores in olfactory associative memory paradigms are reduced in aged flies, the underlying mechanisms were not clear [136, 137]. It turns out that the presynaptic AZs in the MB calyx become larger during ageing, as measured in EM images of the calycal AZs and after super-resolution imaging of the localization of BRP [138]. Interestingly, a similar increase in BRP accumulation was observed in bees as well [139]. The increased size of AZs correlated with augmented SV release. Importantly, a dietary treatment that protects flies from memory loss in ageing [138] could also restore AZ size. Conversely, artificially increased expression of AZ components BRP or RIM-BP in young flies, mimicked the reduced learning performance of aged flies [138]. These data point to the fact that AZs undergo structural changes during ageing (Fig. 3b). They furthermore indicate a role of the presynaptic AZ scaffold in regulating synaptic plasticity during olfactory memory formation and reveal that calycal synapses can modulate memory capacity. Finally, they suggest that re-establishing appropriate presynaptic function might significantly contribute to restoring cognitive impairment associated with ageing.

## Conclusions

Thanks to the relatively small size of its nervous system and to coordinated efforts, the reconstruction of circuits within the brain of *Drosophila* is proceeding at an impressive pace [22, 23]. Large-scale approaches based on electron microscopy are providing maps of every single synapse in large parts of the nervous system. This level of resolution raises now even more clearly the question of stereotypy of neuronal processes and circuits among animals and thus of degrees of freedom in circuit establishment during development- and of plasticity in face of changing experience during adult life.

How much freedom is allowed in setting up connections during development? Answering this question will require a systematic analysis of neuronal morphology and of known connections, ideally at the synaptic level, in a number of animals or at different developmental stages. Such studies are starting to appear [31, 140]. It is possible that certain circuits allow little discrepancy from a basic scheme, while others afford larger degrees of freedom during development. The randomly set up MB calyx would be a good candidate for the latter scenario. What are the mechanisms that control non-stereotyped circuits to attain a balanced level of activity and produce meaningful signals?

In addition to these potentially nervous system-intrinsic levels of control, environmental factors might well play a role in modulating neuron differentiation and circuit assembly. In honeybees, for instance, MG density and size in the adult MB calyx depend on temperature and light experienced by the animals during development [141]. A striking example of control exerted by the growth conditions on the development of the nervous system recently emerged from studies on fate decisions during neuroblast divisions [142–144]. The nutritional state of the animal determines the timing of peaks of production of the hormone Ecdysone [145, 146]. In turn, it is the response to Ecdysone that initiates the fate switch from γ to α’β’ and then to αβ neurons during neuroblast divisions [142].

As detailed in this review, a lot of the work to respond to the questions of to which extent and how experience modulates neuronal circuit development lies ahead of us.

Addressing systematically structural plasticity in the adult nervous system presents a number of even more complex challenges. Faced with circuits and connections that are not stereotyped to the synaptic level or in some cases, as in the calyx, that present little stereotypy, the potential of identifying eventual small modifications will be limited. Fortunately, many of the tools necessary are becoming rapidly available. Fly lines that allow manipulating specifically and independently pre- and postsynaptic partners were recently generated [147, 148] and they will allow to concentrate on reproducible connections. Sophisticated tools for localizing AZ components and some postsynaptic markers have been produced over the past years [10]. Functional imaging in the fly brain can be carried out especially in more accessible brain regions [79, 116, 149]. Activity-dependent gene expression profiles were described in subsets of neurons in the adult fly brain [150]. Functional analysis of the identified genes might in the future shed light on activity-dependent structural refinement processes. With the repertoire of genetic tools in *Drosophila*, a large-scale interrogation of the signals that trigger structural plasticity, its molecular and cell biological mechanisms, as well as the cause-effect relationship between structural changes and their functional and behavioral consequences might be at hand.

## Abbreviations

AL: Antennal lobe
APL: Anterior paired lateral
AZ: Active zone
BN: Bolwig’s nerve
cAMP: Cyclic adenosine monophosphate
DD: Constant darkness
FMRP: Fragile X Mental Retardation Protein
KCs: Kenyon cells
LD: 12 h light/12 h dark cycle
LL: Constant light
LNv: Ventral lateral neurons
LPTCs: Lobula plate tangential cells
MB: Mushroom body
MBONs: MB output neurons
MG: Microglomerulus
NMDAR1: N-methyl-D-aspartate receptor 1
NMJ: Neuromuscular junction
ORs: Odorant receptors
OSNs: Olfactory sensory neurons
PNs: Projection neurons
PPB: Phototaxis preference behavior
SV: Synaptic vesicle
TeTxLC: Tetanus toxin light chain
